# Identification of the Porcine *XIST* Gene and Its Differential CpG Methylation Status in Male and Female Pig Cells

**DOI:** 10.1371/journal.pone.0073677

**Published:** 2013-09-09

**Authors:** Jae Yeon Hwang, Eun Bae Kim, Hakhyun Ka, Chang-Kyu Lee

**Affiliations:** 1 Department of Agricultural Biotechnology, Animal Biotechnology Major, and Research Institute for Agriculture and Life Sciences, Seoul National University, Seoul, Republic of Korea; 2 Department of Food Science and Technology, University of California, Davis, California, United States of America; 3 Department of Animal Products and Food Science, College of Animal Life Sciences, Kangwon National University, Chuncheon, Kangwon-do, Republic of Korea; 4 Division of Biological Science and Technology, Institute of Biomaterials and Institute for Poverty Alleviation and International Development, Yonsei University, Wonju, Kangwon-do, Republic of Korea; University of Bonn, Institut of experimental hematology and transfusion medicine, Germany

## Abstract

*XIST*, a long non-coding RNA, plays an important role in triggering X chromosome inactivation in eutherians, and is used extensively for qualifying stem cells and cloned embryos. However, a porcine *XIST* has not yet been thoroughly identified despite its biological importance in a wide variety of research fields. Here, we present a full-length porcine *XIST* sequence assembled using known sequences (GenBank), RNA-Seq data (NCBI SRA), and PCR/sequencing. The proposed porcine *XIST* gene model encodes a 25,215-bp transcript consisting of 7 exons, including two conserved and two porcine-specific repeat regions. Transcription covering the entire *XIST* region was observed specifically in female cells, but not in male cells. We also identified eight transcription starting sites (TSSs) and evaluated CpG methylation patterns in the upstream (+2.0 kb) and downstream (−2.0 kb) regions. Sixty-seven CG di-nucleotides identified in the target region were considered to be candidate CpG sites, and were enriched in the following two regions: −284 to +53 bp (13 sites) and +285 to +1,727 bp (54 sites) from the selected TSS. Male 5` region of *XIST* (64.5 sites, 96.26%) had a higher level of CpG methylation than female DNA (33.4 sites, 49.85%). Taken together, our results revealed that the porcine *XIST* gene is expressed exclusively in female cells, which is influenced by the lower level of CpG methylation in the putative promoter region compared with male cells. The porcine *XIST* presented in this study represents a useful tool for related research areas such as porcine embryology and stem cell biology.

## Introduction

X-chromosome inactivation (XCI) occurs early in embryo development, and involves silencing one of the X chromosomes in mammalian female cells to compensate gene dosage [1]. Different mammalian species use various strategies to induce XCI during early embryo development [2]. For example, it has been suggested that eutherians except rodents, like humans, employ random inactivation of the X chromosome [2], [3], whereas marsupials and rodents undergo imprinted inactivation of the paternal X chromosome during early embryo development [4], [5]. The X-chromosome inactivation-specific transcript, *XIST/Xist*, which is located at the X-inactivation center (Xic), has been suggested to trigger XCI in eutherian mammals by cis-coating one of the X chromosomes in female mammals [6], [7].

A gene model of *XIST/Xist*, which produces a long, non-coding RNA (lncRNA) [8], [9], showed that *XIST/Xist* consists of large first and last exons with several small exons between these large exons in both humans and mice. Identification of the *XIST* gene was a turning point in research on XCI in various eutherians. For example, the identification of *XIST* allowed for the evaluation of XCI in human, murine, and bovine species during early embryo development [3], [10], [11]. Recently, the importance of *XIST* RNA for early embryo development and embryonic stem cell research has been highlighted. For example, the identification of abnormal expression of *Xist* and X-chromosome-linked genes in a cloned mouse was reported [12], and RNA interference (RNAi)-mediated downregulation of *Xist* during embryo development in cloned mice was shown to dramatically elevate birth rates [13]. These reports suggest an important role for *Xist* in early embryo development. It has also been shown that *Xist* expression is associated with the pluripotency markers Oct4, Sox2, and Nanog, and that lower expression of *Xist* is maintained in undifferentiated mouse embryonic stem cells [14]. *XIST* research has also focused on the status of pluripotency, specifically naïve and primed status, because these two different stem cell types exhibit different patterns of X chromosome inactivation [15]. Therefore, *XIST* has been used as a marker for evaluating the status of pluripotent stem cells [15]. Although the importance of *XIST* has been highlighted in various research fields, only partial *XIST* sequences have been reported in pig [16]. And also, putative gene model resulted from aligning between pig genome and bovine *XIST* was suggested recently, only small fraction compared to the suggested draft gene model was validated just using RT-PCR analysis [17]. A recent paper that defined various genes and RNAs in the pig also identified partial sequences of porcine *XIST*, namely, 84 bp of exon 1 and 126 bp of exon 4 [18]. Although these findings suggest some information about porcine *XIST* RNA, but a large proportion of *XIST* RNA sequence was still unknown. While the identified porcine *XIST* sequences could be used for research related to early embryo development and stem cells, identification of a full *XIST* sequence using gene prediction methods may be difficult [18] because of the low sequence conservation of *XIST* genes among species [19], [20].

The identification of *XIST/Xist* has also led to verification of its promoter region [21], [22] and analysis of the CpG dinucleotide methylation patterns in this region [23]. The *XIST/Xist* promoter region has been suggested to regulate *XIST* expression by recruiting transcription factors [24], and the methylation pattern of the promoter region of *Xist* is highly related to its expression [23]. Analysis of methylation patterns has led to the suggestion that expression of *Xist* in the mouse is controlled by imprinting during preimplantation embryo development [23], [25]. In addition to the identification of CpG sites as differentially methylated regions (DMRs) and factors in CpG sites in the *Xist* promoter region have been used to determine the normality of early embryo development [26]. Specifically, abnormal methylation patterns in the *Xist* promoter have been reported in cloned mouse embryos [26]. Nevertheless, little is currently known about the CpG sites of porcine *XIST*.

In this study, we aimed to identify the candidate porcine *XIST* ncRNA encoding regions using BLAST homology searches. We verified our findings using reverse transcriptase (RT)-PCR, enzyme mapping, sequencing, and RNA-Seq alignment on the whole region. Based on the identified *XIST* sequence, we analyzed the methylation patterns of 67 CpG sites in the region ±2 kb from the transcription start site (TSS) to confirm the DMRs of porcine *XIST*. Our results showed that the porcine *XIST* RNA is nearly 24 kb in length, and contains previously reported partial sequences of porcine *XIST*. The 67 CpG sites that we analyzed exhibited sex-dependent methylation patterns similar to those found in other species.

## Results

### Identification of a Porcine *XIST* Gene Model and its RNA Sequence

**Figure 1:**
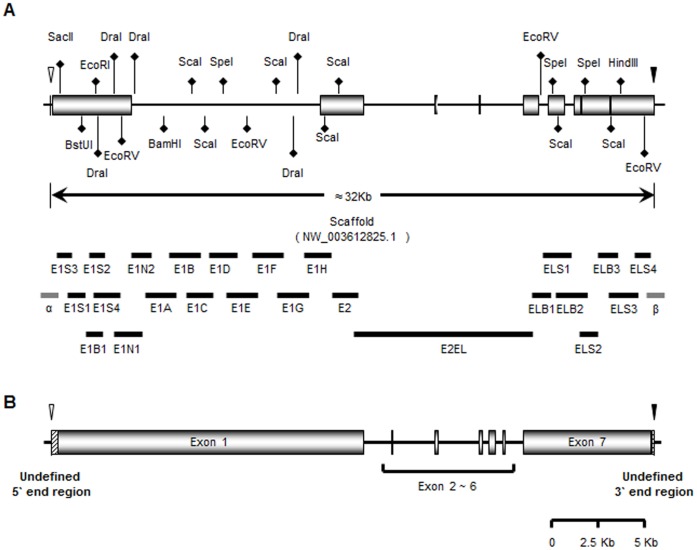
Diagram of the candidate *XIST* expressing region in the pig. (A) Designed *XIST* model and primer sets. Candidate *XIST* ncRNA expression regions on the pig X-chromosome scaffold (NW_003612825.1) determined by BLAST searches are represented by black boxes. Empty and filled arrowheads indicate the 289233^rd^ and 257103^rd^ nucleotides of the scaffold, which were aligned with the transcription start site (TSS) and the last sequence of human and bovine *XIST,* respectively. The designed primer sets are represented as black and gray lines: female-expressed primer sets are represented as black lines and regions that were not expressed in either sex (α and β) are represented as gray lines. Restriction enzymes used to digest each amplicon are shown. The diagram is scaled. (B) Diagram of the identified porcine *XIST* gene model. The porcine *XIST* expressing region was identified by sequencing. Filled rectangles indicate exons, and boxes with diagonal lines indicate undefined regions. Filled and empty arrowheads indicate candidate 5′ and 3′ end regions identified from alignment with human and bovine *XIST*, respectively. The diagram is scaled.

To identify the porcine *XIST* gene and its RNA sequence, we aligned and compared the pig genomic DNA sequence (X-chromosome scaffold, GenBank accession No. NW_003612825.1) with mouse (NC_000086.7), human (NC_000023.10), and bovine *XIST* gene models (AC_000187.1) (Figure S1). The DNA sequence ranging from nucleotides 289,233 to 257,103 in the pig scaffold was well-aligned with the three gene models. Specifically, they matched the TSS and the last nucleotide of the human and bovine *XIST* genes, respectively (Figure 1A and Figure S1). Primer sets (Table S1) were designed to confirm whether the aligned pig sequence (candidate *XIST*) is transcribed in porcine embryonic fibroblast (PEF) cells (Figure 1A). RT-PCR using these primer sets confirmed that the candidate *XIST* gene was transcribed only in female PEFs, and the target regions were not detected in male PEFs (Figure S2). We also confirmed the amplicons by restriction enzyme mapping, which produced patterns consistent with our predictions (Table S2). All of the amplified regions were sequenced by Sanger sequencing, and most of the sequences were consistent with the porcine DNA sequence. However, one amplicon (E2EL) that partially covered the first and last exons of the candidate porcine *XIST* gene was shorter compared with the expected genomic DNA sequence (Figure S2), indicating the presence of introns and exons in the target DNA region. Two other amplicons (E1N1 and E1N2) exhibited longer bands than expected because of a gap (Ns) in the pig scaffold, which was filled with a 626-bp nucleotide sequence from the longer-than-expected amplicons (Figure S3). Finally, the sequenced amplicons were assembled into a porcine *XIST* gene model consisting of two long exons (exon 1 and exon 7) and 5 smaller exons (exon 2 - exon 6) between exons 1 and 7 (Figure 1B). The exons were located between introns with RNA-splicing recognition sequences (GT and AG, Table 1) at their 5′ and 3′ termini, respectively. Our results suggested that the porcine *XIST* gene shares an evolutionarily conserved region among mammals, indicating the reliability of our model.

**Table 1:** Exons and introns information of Porcine *XIST*.

Number	Sequence (5′ to 3′)		Length (bp)
Exon 1	*TATTTCTT	AGACTACT	17,184
Intron1	** **GT**AAGTAC	TTTTAA**AG**	1,460
Exon 2	GGATGAAT	CTCCAAAG	89
Intron2	**GT**GAATCT	TTCTCA**AG**	2,271
Exon 3	GATATTCC	AGAAAAAG	136
Intron3	**GT**AATTTA	TTCTCC**AG**	2,136
Exon 4	ATCTTCCTC	CATCTGAG	209
Intron4	**GT**GGGTAA	TCTTTT**AG**	356
Exon 5	GAAAACAG	TACTCTAG	329
Intron5	**GT**CAGTGG	TTCGGT**AG**	390
Exon 6	CTCCTGAT	AGGATGAA	129
Intron6	**GT**AAGTTG	TCTTCC**AG**	989
Exon 7	TGATTGTC	AAAACTTA	7,139

* 289,233rd nt of NW_003612825.1 scafford sequence was set to first sequence which were one of the identified TSS and matched to blast search in this study.

** Bold sequence, GT/AG, indicate splicing recognition sequence.

### Identification of the 5′ and 3′ end Regions of Porcine *XIST*

The transcript start sites (TSSs) of porcine *XIST* were identified by 5′-RACE PCR using the primer sets (Table S3). The 289,233^rd^ nucleotide of the pig X chromosome scaffold (NW_003612825.1) was expected to be a porcine *XIST* TSS based on sequence alignment with the human and bovine *XIST* 5′ region (Figure S1), which has been suggested to be conserved among mammals [27]. We identified additional 7 TSSs in addition to the expected site (Figure S4). Two TSSs (−5 and −3) were found upstream of the expected TSS, while 5 TSSs (+16, +48, +72, +143, and +211) were located downstream of the expected TSS.

Transcription termination sites were also identified by 3′-RACE PCR. The 257,103^rd^ nucleotide of the pig X chromosome scaffold (NW_003612825.1) was expected to be a porcine *XIST* termination site based on sequence alignment with the human and bovine *XIST* ncRNA sequence. We identified one transcription termination site (257,094^th^ nucleotide) 10 bp downstream of the expected site. The 3′ region was confirmed by RACE analysis, which indicated the presence of an AATAAA poly A-tail signal sequence located 21 bp upstream of the termination site (Figure S4).

### Repeat Sequence Analysis in Porcine *XIST* RNA

**Figure 2:**
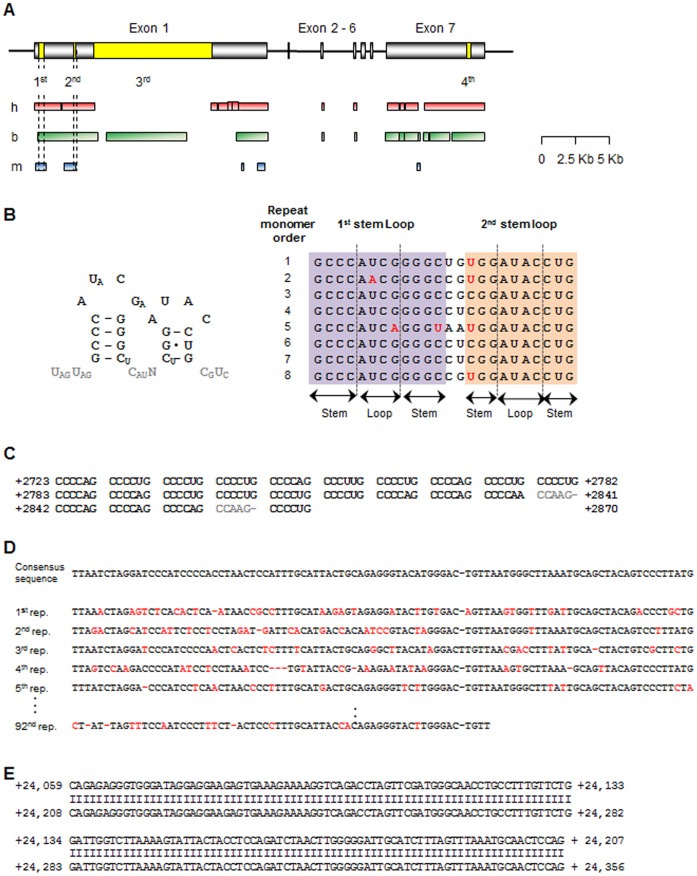
Analysis of repeat sequences in porcine *XIST* RNA. (A) Distribution of repeated sequences in the porcine *XIST* gene model. Gray rectangles indicate exons, and the yellow boxes in the exons indicate repeat regions. The regions having homology with human, bovine, and mouse *XIST/Xist* are represented to red (h), green (b), and blue (m) boxes, respectively. The first and second repeat regions showed similarity with all accessed *XIST/Xist* sequence from other species (dashed lines). The diagram is scaled. (B) The first repeat region in porcine *XIST*. A monomer of the first repeat region in pigs (left panel) share a consensus sequence and a predicted secondary structure of the mouse [28]. Gray text indicates non-conserved sequences. Eight repeated monomers identified in the porcine *XIST* first repeat region (+327 to +695) were aligned with a consensus sequence (Right panel). Red character indicates different sequence compared to the consensus sequence in conserved stem-loop region. (C) The second repeat region (+2,723 to +2,870) is shown, with non-repeat sequences in gray. (D) The third repeat region (+4,302 to +13,102) fraction. The first five repeats and last repeat were aligned with the consensus sequence. Red characters mean the sequences which were not same to consensus sequence. (E) Alignment of the fourth repeat region (+24,059 to +24,356). Two copies of the monomer were perfectly matched.

The repeated sequences in *XIST/Xist* RNA have been suggested to have a function in X chromosome silencing. Therefore, we examined whether these repeated sequences and conserved functional domains are present in the identified porcine *XIST* RNA sequence. Four repeat sequence regions were identified (Figure. 2A). Interestingly, the *Xist* A-repeats comprises copies of a 24-mer consensus sequence known to conserved between humans and mice [9] were detected in the first repeat region of porcine *XIST*. The consensus sequence of the first repeat region was predicted to have a secondary structure containing two stem-loops (Figure 2B), which is the same structure as that of mouse *Xist* [28]. The consensus 24-mer monomer was repeated 8 times in the +327 to +695 region (Figure 2B). The second repeat region contained a cytosine-rich 6-mer repeat that was located in the +2,723 to +2,870 region of the porcine *XIST* RNA as the cysteine-rich repeat-B region of mouse *Xist* [9]. In all, a total of 23 repeats were observed in this region (Figure 2C). We also searched for a consensus sequence of the mouse C-repeat sequence, but did not locate such a sequence in porcine *XIST*. Two novel repeat regions (the third and fourth regions in Figure 2A) were also identified. The third region comprised 91.7 copies of a 96-bp consensus sequence ranging from +4,302 to +13,102 in the porcine *XIST* gene (Figure 2D). The fourth region consisted of two copies of a 149-bp monomer ranging from +24,059 to +24,356 (Figure 2E). Interestingly, consensus sequences in the third region of porcine *XIST* were not present in either human or mouse repeat regions, while the fourth region was only partially aligned to a non-repeating region of human *XIST*. Taken together, the identified porcine *XIST* RNA contained not only conserved sequences, but also porcine-specific repeated sequences, suggesting conserved and hypothetically distinct functions of porcine *XIST*.

### Expression of *XIST* in Other Porcine Tissues

**Figure 3:**
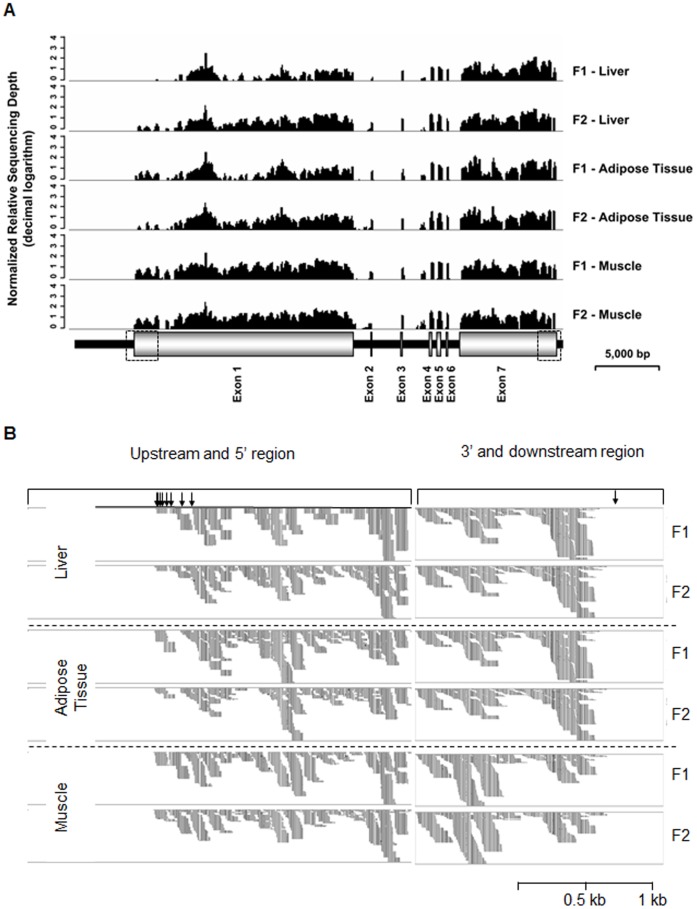
Alignment of Illumina reads to the porcine *XIST* gene model. (A) Alignment of Illumina reads to the porcine *XIST* gene locus. Six Illumina reads from three tissues (liver, abdominal fat, and longissimus dorsi muscle) of two female pigs (F1 and F2) were aligned to the entire porcine *XIST* gene including introns and exons. Small peaks between exon 3 and 4 were identified to be non-specific alignment of reads. The diagram is scaled. The two dotted boxes indicate upstream/5′ and 3′/downstream regions of the *XIST* gene, respectively. (B) Alignment of Illumina reads to upstream/5′ and 3′/downstream regions. Arrows in the upstream and 5′ region indicate the transcription start sites (TSS) of the porcine *XIST* gene identified by 5′ RACE. The arrow at the 3′ and downstream region indicates the end of the porcine *XIST* RNA identified by 3′ RACE.

We assembled a full-length porcine *XIST* RNA sequence by PCR and sequencing and identified its 5′ and 3′ regions. Although assembly and identification of both ends of the RNA strand was performed using PEFs, it was not immediately clear whether *XIST* was also expressed in other porcine tissues in the same manner as our gene model. Thus, to confirm our gene model and verify the expression of *XIST* in other tissues, Illumina sequence reads from three different porcine tissues–liver, abdominal fat, and the longissimus dorsi muscle–of two female pigs were aligned with our porcine *XIST* gene model (Figure 3A). The Illumina sequence reads were well aligned onto the most of the region of the model, including the 5′ and 3′ regions (Figure 3 and S5), while none of the Illumina reads were aligned to the upstream/downstream regions of the assembled *XIST* RNA (Figure 3B). Thus, our results suggested that *XIST* is expressed in other porcine tissues as described above.

### Analysis of Methyl CpG Sites in the Upstream Region of Porcine *XIST*

**Figure 4:**
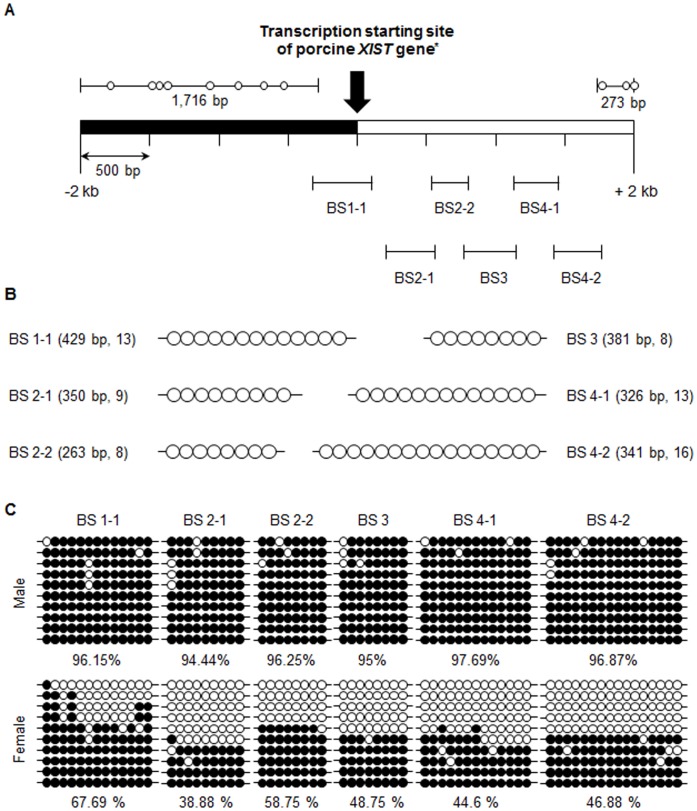
Diagram of the CpG sites located in the ±2 kb region of the transcription start site of porcine *XIST* and the methylation status of *XIST* CpG sites in male and female porcine embryonic fibroblasts. (A) One of the transcription start sites (TSS) identified by 5`-RACE-PCR was set as a +1 (asterisk, 289233^rd^ nucleotide of NW_003612825.1). Black and empty bars indicate regions upstream and downstream of the TSS, respectively. Methylation of CpG sites in six regions (BS1-1, BS2-1, BS2-2, BS3, BS4-1, and BS4-2) was analyzed. The diagram is scaled. (B) Profiles of the six target regions. Each circle represents a single CG dinucleotide identified in the amplified region. The length of each amplicon was 250 bp –450 bp and contained between 8–16 CG dinucleotides. The diagram is not scaled. (C) The methylation status of 67 *XIST* CpG sites were analyzed in male and female embryonic fibroblasts. Each circle indicates a CpG dinucleotide. Filled and empty circles represent methylated and unmethylated CpG dinucleotides, respectively. The horizontal line represents one individual clone.

After identifying the TSSs of the porcine *XIST* gene, we next analyzed the methylation status of the CpG sites upstream of the *XIST* TSS. The search for CG dinucleotides was conducted using the ±2 kb region of the first porcine *XIST* sequence (289,233^rd^ nucleotide of NW_003612825.1, Figure S4), which identified 78 CG dinucleotides, 67 of which were densely located between nucleotides −284 and +1,727 (Figure 4A). The methylation statuses of the 67 CpG sites in both male and female embryonic fibroblasts were analyzed using six different primer sets (Table S4, and Figure 4B). With the exception of five CpG sites, the analyzed regions were over 90% methylated in male PEFs, and the total methylation rate for male PEFs was 96.26% (Figure 4C). However, in female PEFs, although five CpG sites on the BS1-1 region were highly methylated (>70%), the remaining CpG sites were methylated at a rate of 40∼60%. Thus, the total methylation rate of the 67 CpG sites in female PEFs was 49.85% (Figure 4C). The results suggested the analyzed CpG sites were differentially methylated between the male and female.

## Discussion

To the best of our knowledge, this study is the first to report a gene model of porcine *XIST* that includes a ncRNA sequence and the CpG methylation status of its regulatory regions. The purpose of XCI is to balance the expression level of X-chromosome-linked genes between male and female eutherian mammals, occurs early during embryo development [1], [2], [29]. The initiation of XCI is induced by cis-binding of *XIST/Xist* onto the X chromosome [6], [7]. Interestingly, among the different strategies for XCI in eutherian mammals [2], [30], all appear to use *XIST* as a trigger for XCI. The importance of *XIST/Xist* has been a focus of not only XCI research, but also, more recently, stem cell research [14], [15] and embryo cloning research [12], [13]. Previous studies have indicated that *XIST* is associated with stem cell differentiation [14] and abnormalities of cloned embryos in the mouse [12]. Despite the importance of *XIST* and XCI in a number of research fields, only a partial sequence of porcine *XIST* has been previously reported [16]–[18]. Add to this, the porcine *XIST* expression and methylation status on its candidate CpG sites were analyzed in our previous report comparing porcine embryonic stem cells from different origins and induced pluripotent stem cells. However, the information about porcine *XIST* used for the study was restricted and kinds of putative ones, so identification of porcine *XIST* whole sequence and analysis of its regulating sites should be accessed to perform more accurate and detailed *XIST* analysis in pig pluripotent cells and embryos. This led us to generate a porcine *XIST* gene model, including its ncRNA sequence, and to examine the CpG methylation status of its regulatory regions.

Sequence alignment was used to identify candidate porcine *XIST* exons, which showed not only an evolutionarily conserved *XIST* gene model among mammals, but also species-specific sequence divergence. Several evolutionarily conserved regions, such as repeated sequences among mammalian *XIST* orthologs, have been previously reported [9], [27]. Indeed, the A-F regions are regarded as functional domains of *Xist* [9], [28], [31], some of which were present in our model of porcine *XIST*. On the other hand, divergence of *XIST/Xist* sequences among species due to rapid ncRNA evolution has been noted [19], [20]. Consistent with this observation, porcine *XIST* also exhibited sequence divergence in some regions compared with other mammalian *XIST/Xist* genes, and showed varying sequence similarity depending on the compared target species. However, certain regions, such as the 5′ region of exon 1, were highly conserved among species, as shown in Figure S1.

A previous study suggested that *XIST/Xist* originated from a protein-coding gene (*Lnx3*) and transposable elements [27], and specifically that the 5′ region of exon 1 and exons 5 through 7 of the consensus *XIST* sequence arose from a protein-coding gene. In this study, we showed that the same regions of human and bovine *XIST* were well-aligned to that of porcine *XIST* (Figure S1), suggesting that the highly conserved domains originating from the proposed protein-coding gene survived in pigs during evolution, and may have a role in porcine *XIST* function. In contrast, some regions originating from transposable elements are suggested to have integrated into *XIST* after mammalian taxa divergence, resulting in species-specific sequence variation among eutherian *XIST*/*Xist* [27]. Such sequence variation may account for the presence of porcine-specific regions that did not align to the *XIST/Xist* sequences of other species, and which may also contribute to species-specific modes of *XIST* function during XCI. Together, these evolutionarily properties of *XIST/Xist* support the reliability of the porcine *XIST* expression region.

Most of the RT-PCR target regions, except the α and β regions, were amplified exclusively in female porcine cells (Figure 1 and S2), which was highly consistent with previous observations in other mammals [2], [29], [32], [33]. In particular, primer pairs that amplified regions that were not selected as part of the first candidate model (E1N1 to E1H in Figure 1A) exhibited female-specific expression (Figure S2). These regions may have evolved in a porcine-specific manner as suggested previously [27], and may contribute to a novel role of *XIST*-associated XCI distinct from that in other species.

In the mammalian *XIST/Xist* gene model, the first and last exons are much longer than the central exons [8], [9]. As expected, the E2EL amplicon contained 5 small exons between the first and last exons, consistent with other species (Figure 1B). Furthermore, the identified exon 4 was conserved in pigs (Figure S1 and [34]). These results suggested that the porcine *XIST* ncRNA shares a common model with other mammalian *XIST/Xist* genes.

The identified porcine *XIST* gene model covered a previously annotated partial porcine *XIST* sequence in the 3′ region of *XIST* (GenBank accession Nos. AJ429140 and EF619477.1). In addition, the recently annotated partial exon 1 (nannotator00001430) and exon 4 (nannotator00001519) sequences were also located in the expected porcine *XIST* exons (http://rth.dk/resources/rnannotator/susscr102/). The presence of a previously reported partial porcine *XIST* expression region on the defined *XIST* gene supports the reliability of our model of porcine *XIST.*

The repeat sequences in *XIST/Xist* have been suggested to have functional roles in XCI [8], [9]. Interestingly, some repeat regions were detected in the porcine *XIST* ncRNA sequence. The A repeat, which consists of 7.5 repeats of a 24-bp sequence, forms two stem-loop in mouse, and has been known to regulate processes essential for X chromosome silencing [28]. Interestingly, Wutz and colleagues showed that a mutated A region allele results in a failure to induce X chromosome silencing in a stem cell model without affecting *Xist* stability and localization [28]. The identified porcine *XIST* 5′ region included a total of 8 copies of the 24-bp sequence (Figure 2A and 2B), and this region was well-aligned to the 5′ region of mouse *Xist* (Figure 2A). This result suggested that, similar to the A-repeat in mice, the conserved first repeat region in porcine *XIST* may be involved in silencing X chromosomes, although this possibility needs to be further analyzed. Another repeat region, the C-repeat, which consists of 14 copies of an approximately 100-bp sequence in mice, has been suggested to be involved in *Xist* localization during XCI [31]. However, targeting of the C region with Locked nucleic acids (LNAs) does not affect the localization of human *XIST* [31], which has only one copy of the C-repeat [9], [34]. The porcine *XIST* sequence has a sequence similar to that of the human C-repeat sequence (49 bp downstream of the second repeat region), while only one copy was found in the pig. Thus, our results may suggest that porcine *XIST* has a novel mechanism of regulating *XIST* localization on the X chromosome compared with the mouse. Two other repeat regions of porcine *XIST* (regions 3 and 4 in Figure 2A) were identified as candidate functional domains. In particular, the third repeat region consisted of a highly repeated sequence (91.7 copies, Figure 2D) and was found to be well-aligned only with the bovine *XIST* sequence (Figure 2A). The D-repeat has been suggested to be the largest repeat, and is commonly found in exon 1 of *XIST/Xist* in many species [27], [34]. The D-repeat is hyper-variable with respect to both its sequence and length among species [34]. Thus, we hypothesized that the third repeat in porcine *XIST* may be either a D region or a porcine-specific repeat. While we identified four repeat regions in porcine *XIST*, their biological roles need to be clarified in future studies.

The alignment of Illumina reads (NCBI SRA accession numbers: SRX054582 - SRX054587) with our *XIST* gene model clearly demonstrated that the identified regions were expressed in other tissues according to the same model and that transcription start and termination sites at the 5′ and 3′ regions, respectively, were consistent among different porcine cell types (Figure 3 and Figure S5). Taken together, our results suggest that our model of the porcine *XIST* gene and the estimated 5′/3′ regions is reliable.

The promoter of *XIST/Xist* is important for regulating differential expression between female and male eutherians. Two representative *XIST* promoters, P1 and P2, have been reported. P1, a minimal promoter, is located close to the TSS [21], [22], while P2 is located nearly 1 kb from P1 [27], [35]. In our study, there was a region of high CG dinucleotide density from the −284^th^ nucleotide to +53^rd^ nucleotide and from the +285^th^ nucleotide to +1727^th^ nucleotide. The distribution patterns of CpG sites of porcine *XIST* were similar to P1 [21], [22] and P2 [27], [35] of other species. A previous study demonstrated that the *Xist* promoter is highly methylated on active X chromosomes in somatic cells, while inactive X chromosomes do not have a methylated promoter region [23]. Likewise, male porcine somatic cells carrying an active X chromosome were also highly methylated at *XIST* regulatory regions, while female porcine somatic cells carrying both active and inactive X chromosomes displayed a half methylation pattern (Figure 4C). Our results were consistent with those of previous reports, and support the hypothesis that the analyzed porcine CpG sites may be regulatory regions of *XIST* expression. Several transcription factors, such as CTCF, YY1, and TBP1, are known to regulate *XIST/Xist* expression at promoter regions [21], [22], [24], [36]. Interestingly, we confirmed a TBP1 consensus sequence in the candidate porcine P1 region (TTAAAG, −30 to −25), suggesting that TBP1 may be a regulator of porcine *XIST* expression. The presence of this TBP1 consensus sequence in 30bp upstream of one of the TSS, the 289,233^rd^ nucleotide of the pig X chromosome scaffold (NW_003612825.1), suggest that the TSS which was applied to the first sequence of porcine *XIST* in this study is most reliable TSS of the porcine *XIST* RNA. Although we tried to find CTCF-binding sites in *XIST* gene and its upstream region, because of its diverse binding sites, it was hard to determine presence of suitable sites for CTCF binding. The YY1 of which consensus sequences, CCGCCATNTT, is also present in near the *XIST* TSS we identified (CCGCCATATT, −6 to +4), but it is unclear this region is actually recruited as an YY1 binding and functional site. So it should be analyzed these transcription factors are also functional in porcine *XIST* expression as in other species *XIST/Xist* expression. So the exact porcine *XIST* promoter regions also need to be further defined in order to clarify the relationship between CpG methylation and transcription factor binding on *XIST* expression using like chromatin immunopriciptation (Chip) - assay.

In this study, we identified the entire coding region of porcine *XIST* using sequence alignment, RT-PCR, Sanger sequencing, restriction enzyme mapping, and Illumina alignment. We proposed a porcine *XIST* ncRNA 25,215 bp in length and comprising seven exons (Table 1, GenBank accession No. KC753464). We also examined CpG methylation in the regulatory regions of porcine *XIST*. The porcine *XIST* gene model and CpG methylation profile reported here may be important for understanding the mechanism of XCI in pigs. Further studies will be needed to determine the implications of our model on the stem cell biology and embryology of pigs.

## Materials and Methods

The pig experimental processes were performed strictly in accordance with the Guide for Care and Use of Research Animals in Teaching and Research. The experiment was approved by the Institutional Animal Care and Use Committee of Yonsei University, Wonju, Republic of Korea.

### BLAST Search

The entire pig genomic sequence (*Sus scrofa* genome, version 10.2) was compared to the exons of mouse *Xist* and human and bovine *XIST* (Accession Nos: NC_000086.7, NC_000023.10, and AC_000187.1, respectively) using BLAST. Regions on the pig X chromosome that gave an alignment score of over 300 and that matched at least two *Xist* or *XIST* sequences from other species were considered to be candidate encoding regions of the porcine *XIST* ncRNA.

### Sample Preparation, RNA Extraction, and Reverse Transcription

The porcine embryonic fibroblasts (PEF, mixed breed) were obtained from the days post coitus (dpc) = 27 fetuses. Cultured male and female PEFs (passage = 5) were used in this study. PEF was cultured in Dulbecco’s modified Eagle’s medium (DMEM, Gibco Invitrogen, Carlsbad, CA) supplemented with 10% fetal bovine serum (FBS; collected and processed in Canada; Hyclone, Logan, UT), 2 mM glutamax (Gibco Invitrogen, Carlsbad, CA), 0.1 mM ß-mercaptoethanol (Gibco Invitrogen, Carlsbad, CA), and 1× antibiotic/antimycotic (Gibco Invitrogen, Carlsbad, CA) as previously described methods [37]. Total RNA was extracted from cultured PEFs using TRIzol (Invitrogen, Carlsbad, CA, USA) according to the manufacturer’s protocol. Extracted RNA was diluted with sterile DEPC-treated water (pH = 8.0) and treated with recombinant DNase I (Takara Bio, Otsu, Shiga, Japan). DNase-treated RNA samples (2.5 µg) were converted to cDNA in 20 µl final volume using Superscriptase III (Invitrogen Carlsbad, CA, USA) following the manufacturer’s instrument.

### PCR Amplification and RACE-PCR of 5′- and 3′- Region

One-micro litter of synthesized cDNAs was subjected to PCR using the primers listed in Table S1. PCR amplification was carried out using a 2× PCR master mix solution (iNtRON Bio Technology, Seongnam, Gyeonggi, Korea) containing 0.5 µM of each primer set in 10 µl reaction volume. Detailed amplification conditions were described in supplementary information. All 5′- and 3′-region amplifications were performed using RACE-PCR with the 5′-RACE Core Set and 3′-RACE Core Set (Takara Bio, Otsu, Shiga, Japan), respectively. The process for synthetizing cDNA was carried out in accordance with the manufacturer’s instructions and the amplification of synthesized cDNA samples were performed with modifications (see Material and method S1). Reverse-transcrition and amplification was performed using the primer pairs listed in Table S2.

### Enzyme Cutting, Cloning, and Sequencing

Gel-extracted amplicons were purified using a commercial spin column (MEGAquick-spin™ Total Fragment DNA Purification Kit; iNtRON Bio Technology, Seongnam, Gyeonggi, Korea) for restriction enzyme mapping and cloning. Each purified PCR product was incubated with 2 U of the appropriate restriction enzyme listed in Table S3 at 37°C for 2 hours. Amplicons were then cloned into the pGEM-T Easy vector (Promega, Madison, WI, USA) and transformed into *E. coli* (DH5α strain, Novagen, Madison, WI, USA). Plasmid samples were prepared using commercial spin columns (DNA-spin™ Plasmid DNA Purification Kit; iNtRON Bio Technology, Seongnam, Gyeonggi, Korea. Purified plasmid samples were sequenced with an ABI PRISM 3730 DNA Analyzer (Applied Biosystems, Foster, CA, USA) and compared with genomic scaffold sequences.

### gDNA Extraction and Bisulfite Sequencing

Extraction of gDNA was performed using the G-spin Genomic DNA Extraction Kit for cell/tissue (iNtRON Bio Technology, Seongnam, Gyeonggi, Korea) according to the manufacturer’s instructions. Extracted gDNA was treated with bisulfite using the EZ DNA Methylation-Gold Kit (Zymo Research, Irvine, CA, USA) for methylation analysis of CpG dinucleotides according to the manufacturer’s protocol. Bisulfite treated gDNA was amplified with primer pairs listed on the Table S4 and a total of 67 CpG sites were analyzed following the condition described in supplementary information. Amplicons were cloned and sequenced as described above for enzyme cutting, cloning, and sequencing.

### Illumina Sequences, Sequence Alignment, and Alignment Visualization

We used released Illumina sequences from one pair of female pig full-siblings (240 days old, breed: White Duroc X Erhualian F2) stored in the NCBI Sequence Read Archive (SRA) database (Accession Nos. SRX054582 - SRX054587). The reads contained paired-end 90-bp sequences (200-bp insert) generated using the Illumina HiSeq 2000 platform for three different tissues–liver, abdominal fat, and the longissimus dorsi muscle. Custom Perl scripts were used to enrich for higher quality (Q20) reads, which were subsequently aligned to *XIST* RNA sequences assembled manually using Bowtie2 [38]. Aligned reads were sorted using SAMtools [39] and visualized with Tablet [40].

## Supporting Information

Figure S1 ***XIST*** **/** ***Xist*** **homologue analysis of the pig genome by BLAST search.** (A) Human *XIST*, (B) bovine *XIST*, and (C) Mouse *Xist* (GenBank accession Nos. NC_000023.10, AC_000187.1, and NC_000086.7 respectively) models are represented as black boxes (exons) and black lines (introns). Black dashed lines under each *XIST*/*Xist* gene indicate the pig X-chromosome scaffold (NW_003612825.1). Filled arrowheads indicate the transcription start sites (TSS) of each *XIST*/*Xist* gene. Empty arrowheads present on the defined regions of the counterparts of *XIST/Xist* gene indicate candidate porcine *XIST* TSSs based on BLAST alignment (289233^rd^ nucleotide of the NW_003612825.1 scaffold). Arrows indicate the terminus of the *XIST/Xist* gene and the candidate last-sequence of the porcine *XIST* gene identified by aligning human/bovine *XIST* RNAs to the pig genome sequence. Colored lines are homologue regions in each *XIST*/*Xist* gene, and the colored boxes are the counterparts of the lines. Each color represents an alignment score. The diagram is scaled.
(TIF)

Figure S2 **RT-PCR analysis of each candidate region of** ***XIST*** **PCR amplicon expression.** (A) Amplified PCR products of designed primer pairs. Female PEF cDNA (1), male PEF cDNA (2), female PEF gDNA (3) and male PEF gDNA (4) were used for amplification using each designed primer pairs and distilled water (5) was used as a negative control. The asterisk, filled arrowhead, empty arrowhead, and arrow represent the 2000, 1500, 1000, and 750 bp DNA bands of the ladder, respectively. (B) Summary heat map for PCR detection of porcine *XIST* RNA and genomic DNA sequences. Filled gray boxes indicate the presence of PCR target regions. White blank boxes indicate the absence of PCR target regions. The E2EL region was detected only in cDNA templates from female cells.
(TIF)

Figure S3 **Identified gap sequence in the pig X-chromosome scaffold, NW_003612825.1.** A gap sequence region was amplified with primer pairs designed to span the gap region. An unknown region nearly 500 bp longer than the expected length (100 bp) was observed. The gray-boxed sequence (TTTAAA) indicates a DraI restriction site.
(TIF)

Figure S4 **Identification of transcription start sites and the termination site of porcine** ***XIST*** **.** (A) Transcription start sites (TSSs) of the porcine *XIST* gene were identified by 5′ RACE-PCR. Eight putative TSSs were confirmed (red color characters), and the asterisk-marked sequence was determined to be the candidate TSS based on a BLAST homology search (289,233^rd^ nucleotide of the pig X-chromosome scaffold sequence, NW_003612825.1). The TSS (289,223^rd^ nucleotide of NW_003612825.1, asterisk-marked “T”) was set as +1. (B) The last sequence of porcine *XIST* was defined by 3′ RACE-PCR. The italic sequence (“T”, 257,103^rd^ nucleotide of NW_003612825.1) is expected to be the last sequence based on BLAST alignment. The sequence marked with an asterisk (“A”, 257094^th^ nucleotide of NW_003612825.1 and 32,817^th^ nucleotide from TSS) indicates the last sequence identified by 3′ RACE-PCR. The gray-boxed region (AATAAA) represents one of the poly A-tail signal sequences.
(TIF)

Figure S5 **Illumina sequencing coverage of porcine** ***XIST*** **mRNA expression.** The entire region of *XIST* mRNA and upstream/downstream regions was used as a reference sequence for Illumina sequence alignment to show mRNA sequence distribution. The Illumina RNA-Seq was obtained from three tissues of different female individuals (F1 and F2). Sequencing depth was normalized using the total number of aligned reads. Several extreme peaks were observed because of sequence similarity among repeated sequences within the *XIST* gene. This peak could be lowered by increasing alignment stringency.
(TIF)

Table S1 The List of primer pairs sequence for reverse transcription PCR.
(DOCX)

Table S2 List of restriction enzymes used for each amplicon and the predicted sizes of the digested fragments.
(DOCX)

Table S3 List of primer pair sequences for 5`- and 3`- RACE-PCR.
(DOCX)

Table S4 List of primer pairs for bisulfite sequencing.
(DOCX)

Material and method S1 **PCR amplification condition.**
(DOCX)
