# Supplementary figures and images for: Identification of the Porcine XIST Gene and Its Differential CpG Methylation Status in Male and Female Pig Cells
Source: PLoS One. 2013 Sep 9;8(9):e73677. doi: 10.1371/journal.pone.0073677 (PMC3767593; doi:10.1371/journal.pone.0073677)

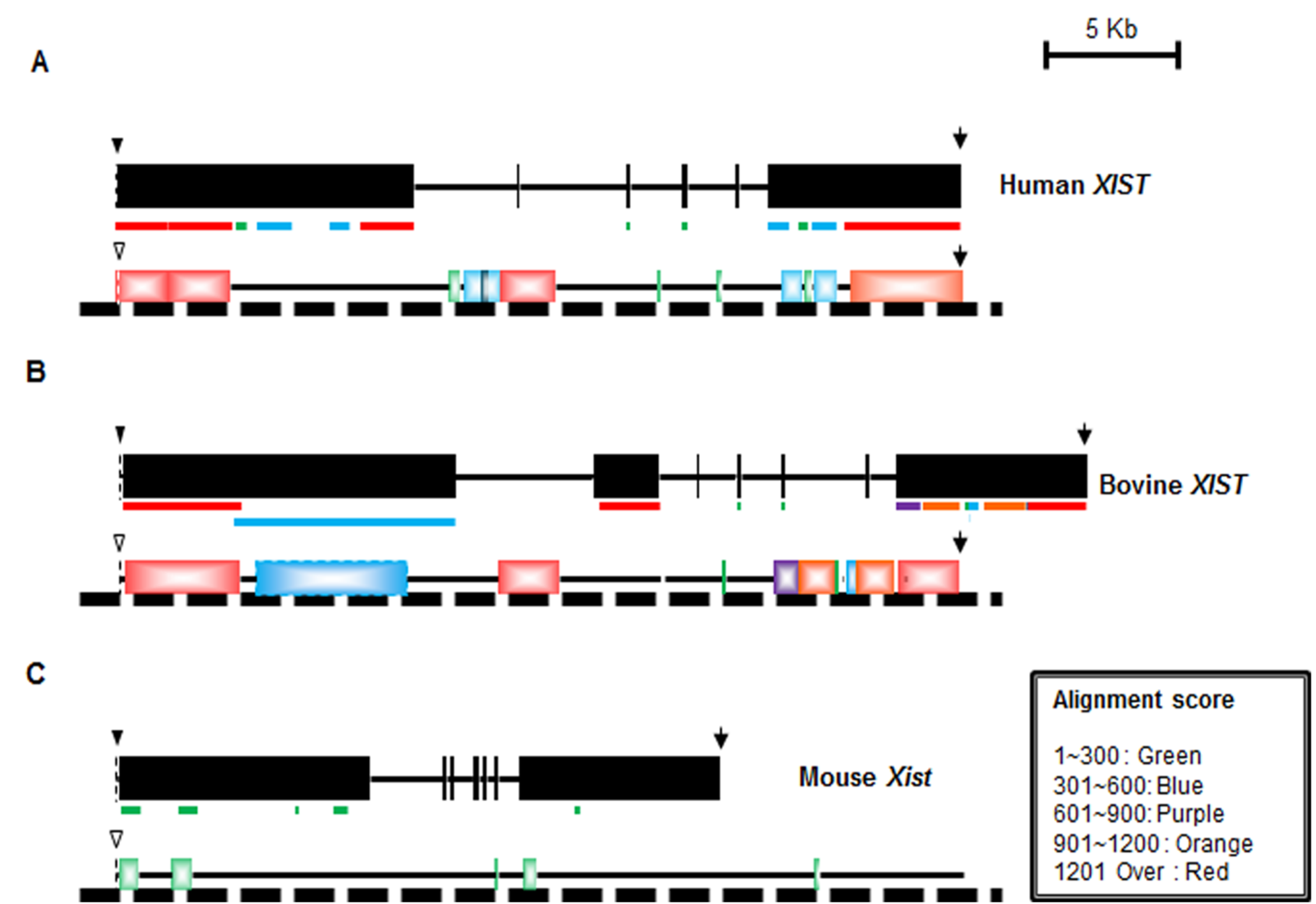

Supplement: Figure S1 — XIST / Xist homologue analysis of the pig genome by BLAST search. (A) Human XIST, (B) bovine XIST, and (C) Mouse Xist (GenBank accession Nos. NC_000023.10, AC_000187.1, and NC_000086.7 respectively) models are represented as black boxes (exons) and black lines (introns). Black dashed lines under each XIST/Xist gene indicate the pig X-chromosome scaffold (NW_003612825.1). Filled arrowheads indicate the transcription start sites (TSS) of each XIST/Xist gene. Empty arrowheads present on the defined regions of the counterparts of XIST/Xist gene indicate candidate porcine XIST TSSs based on BLAST alignment (289233rd nucleotide of the NW_003612825.1 scaffold). Arrows indicate the terminus of the XIST/Xist gene and the candidate last-sequence of the porcine XIST gene identified by aligning human/bovine XIST RNAs to the pig genome sequence. Colored lines are homologue regions in each XIST/Xist gene, and the colored boxes are the counterparts of the lines. Each color represents an alignment score. The diagram is scaled. (TIF) [file pone.0073677.s001.tif]

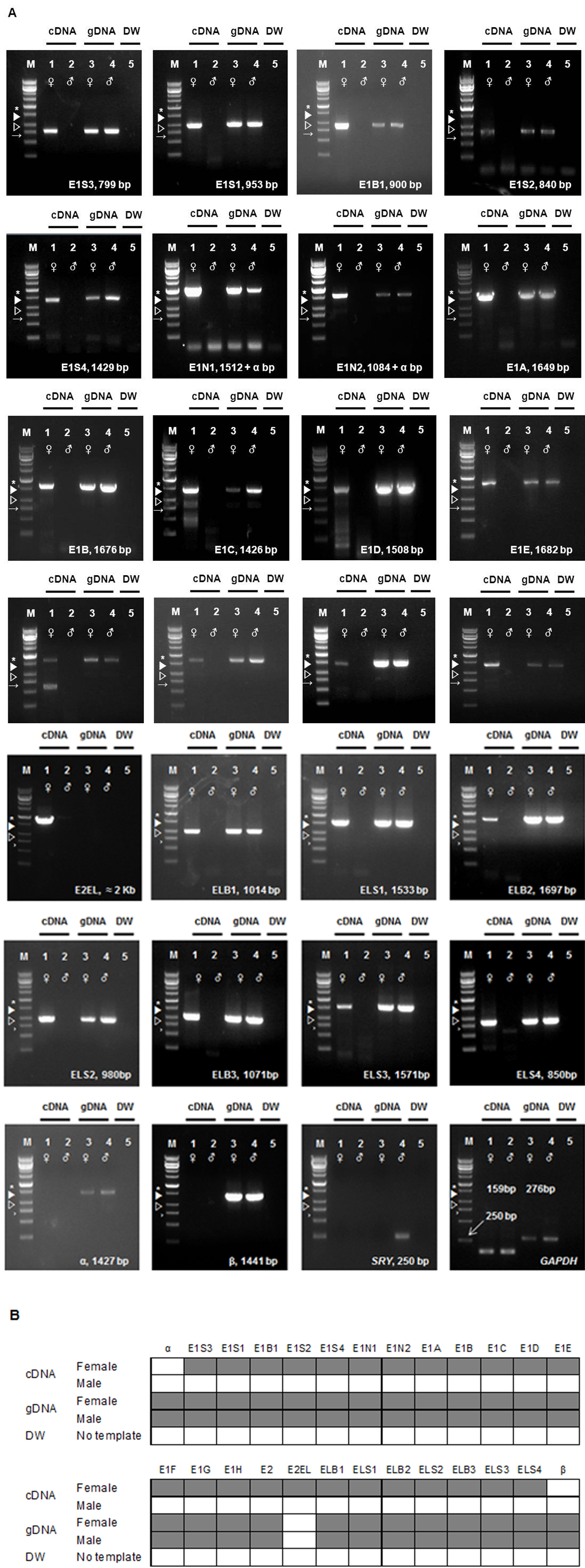

Supplement: Figure S2 — RT-PCR analysis of each candidate region of XIST PCR amplicon expression. (A) Amplified PCR products of designed primer pairs. Female PEF cDNA (1), male PEF cDNA (2), female PEF gDNA (3) and male PEF gDNA (4) were used for amplification using each designed primer pairs and distilled water (5) was used as a negative control. The asterisk, filled arrowhead, empty arrowhead, and arrow represent the 2000, 1500, 1000, and 750 bp DNA bands of the ladder, respectively. (B) Summary heat map for PCR detection of porcine XIST RNA and genomic DNA sequences. Filled gray boxes indicate the presence of PCR target regions. White blank boxes indicate the absence of PCR target regions. The E2EL region was detected only in cDNA templates from female cells. (TIF) [file pone.0073677.s002.tif]

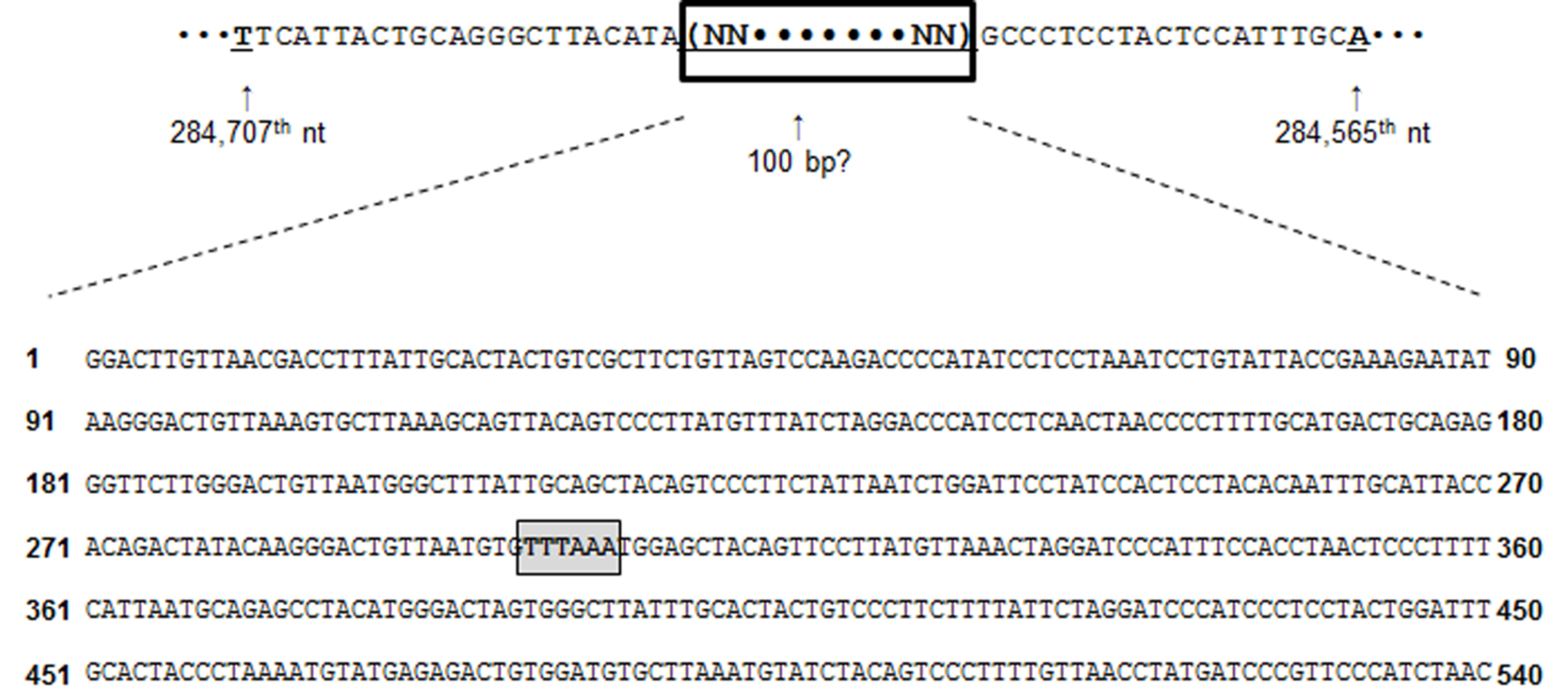

Supplement: Figure S3 — Identified gap sequence in the pig X-chromosome scaffold, NW_003612825.1. A gap sequence region was amplified with primer pairs designed to span the gap region. An unknown region nearly 500 bp longer than the expected length (100 bp) was observed. The gray-boxed sequence (TTTAAA) indicates a DraI restriction site. (TIF) [file pone.0073677.s003.tif]

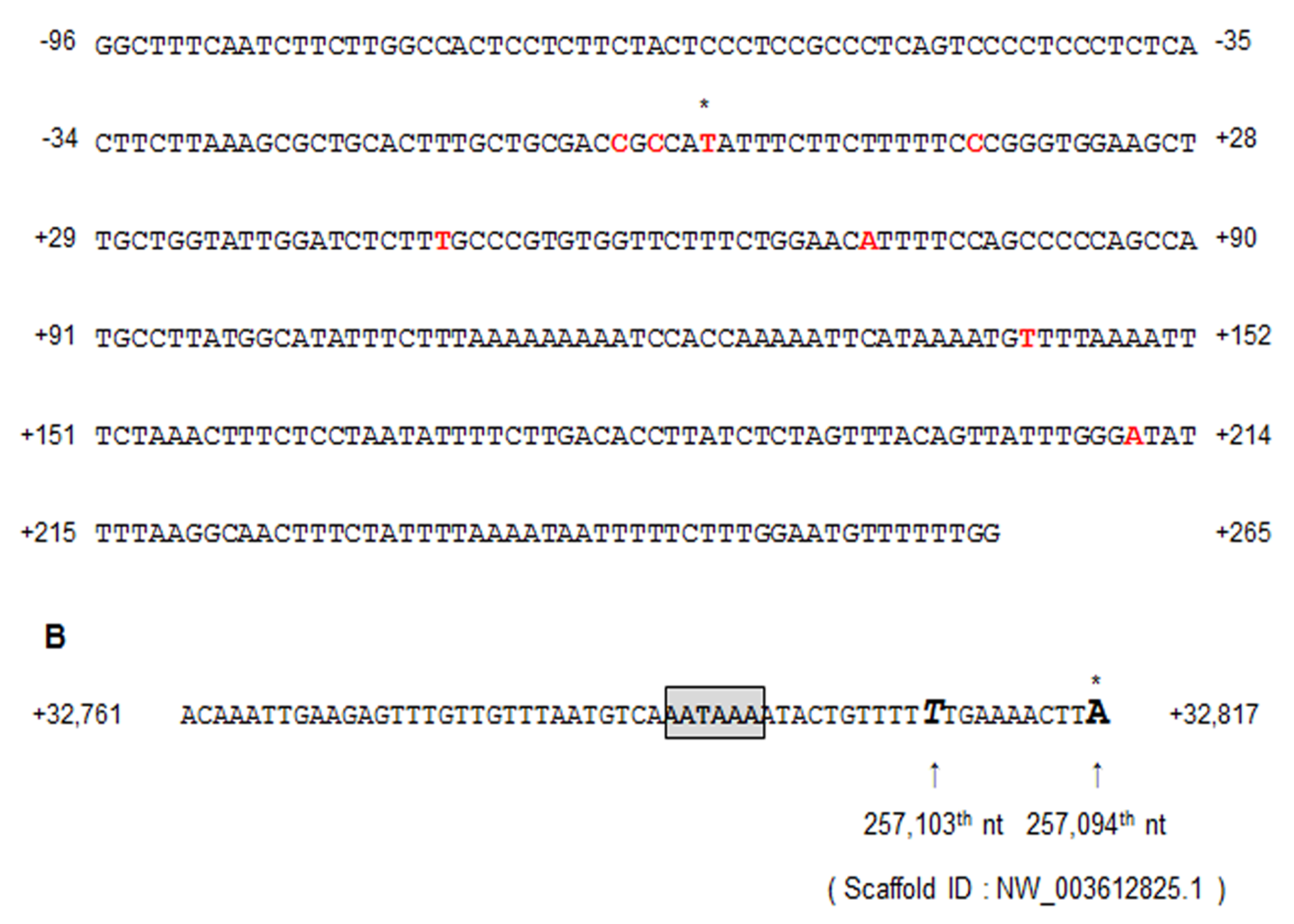

Supplement: Figure S4 — Identification of transcription start sites and the termination site of porcine XIST . (A) Transcription start sites (TSSs) of the porcine XIST gene were identified by 5′ RACE-PCR. Eight putative TSSs were confirmed (red color characters), and the asterisk-marked sequence was determined to be the candidate TSS based on a BLAST homology search (289,233rd nucleotide of the pig X-chromosome scaffold sequence, NW_003612825.1). The TSS (289,223rd nucleotide of NW_003612825.1, asterisk-marked “T”) was set as +1. (B) The last sequence of porcine XIST was defined by 3′ RACE-PCR. The italic sequence (“T”, 257,103rd nucleotide of NW_003612825.1) is expected to be the last sequence based on BLAST alignment. The sequence marked with an asterisk (“A”, 257094th nucleotide of NW_003612825.1 and 32,817th nucleotide from TSS) indicates the last sequence identified by 3′ RACE-PCR. The gray-boxed region (AATAAA) represents one of the poly A-tail signal sequences. (TIF) [file pone.0073677.s004.tif]

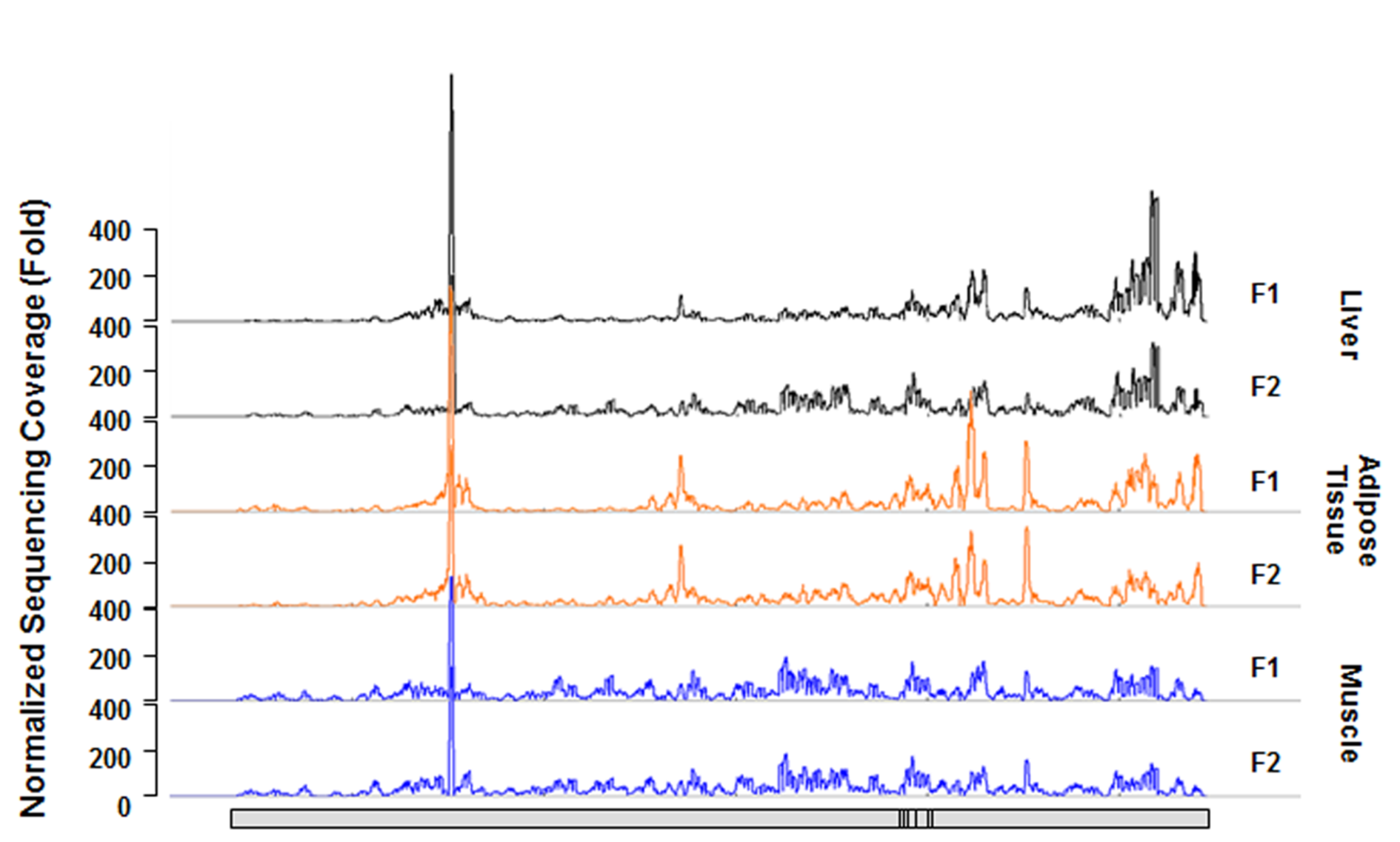

Supplement: Figure S5 — Illumina sequencing coverage of porcine XIST mRNA expression. The entire region of XIST mRNA and upstream/downstream regions was used as a reference sequence for Illumina sequence alignment to show mRNA sequence distribution. The Illumina RNA-Seq was obtained from three tissues of different female individuals (F1 and F2). Sequencing depth was normalized using the total number of aligned reads. Several extreme peaks were observed because of sequence similarity among repeated sequences within the XIST gene. This peak could be lowered by increasing alignment stringency. (TIF) [file pone.0073677.s005.tif]
