# Supplementary material for: Identification of the Porcine XIST Gene and Its Differential CpG Methylation Status in Male and Female Pig Cells
Source: PLoS One. 2013 Sep 9;8(9):e73677. doi: 10.1371/journal.pone.0073677 (PMC3767593; doi:10.1371/journal.pone.0073677)
Supplement: Table S1 — The List of primer pairs sequence for reverse transcription PCR. (DOCX) [file pone.0073677.s006.docx]

| Table S1. The List of primer pairs sequence for reverse transcription PCR | | |  |
| --- | --- | --- | --- |
| ^*^Primer/Gene (Accession. Nos) | Sequence (5` → 3`) | Tm (°C) | †Expected length (bp) |
|  |  |  |  |
| α | F : TACACTTTCCAGTTCCGCCA | 64 °C | 1427 bp |
|  | R : AGGTATCCACAGCCCCGA |  |  |
| E1S3 | F : TTTTCCCCCTCCTTTTCTGT | 58 °C | 799 bp |
|  | R : TGACCCCGTTTTTCCACCTC |  |  |
| E1S1 | F : AGGCTGGGGAGGAAAGATGG | 60 °C | 953 bp |
|  | R : GGTGGTAATGCTGGAGGGGA |  |  |
| E1B1 | F : CAGACAGTTAGTGGAGGATGGAA | 60 °C | 899 bp |
|  | R : AAGGATGGGAACAAAGCAAGA |  |  |
| E1S2 | F : AACAGGTGGCGGAAGAGGAA | 64 °C | 840 bp |
|  | R : AAACAGGAGTGGGACAGGGG |  |  |
| E1S4 | F : AGTGGGTTTTCATTTTGGG | 60 °C | 1429 bp |
|  | R : GGGTCTTATCTGGTAGGCA |  |  |
| E1N1 | F : GGCATCCTACCATTTTTACCCTCT | 66 °C | 1512 bp |
|  | R : AAAGTCCCTTGTATCCTCCCTTG |  |  |
| E1N2 | F : GTCTCACACTCAATAACCGCCT | 66 °C | 1084 bp |
|  | R : TACTCTCAGCAGCCGTCCTAAA |  |  |
| E1A | F : AGAAGAAAGGGTGGGGGAAAAAC | 66 °C | 1649 bp |
|  | R : ACGGGAGGGGTCAGTAGAGCA |  |  |
| E1B | F : ATTCCCGTTCCCTTACTCGTTTT | 66 °C | 1676 bp |
|  | R : TCCCTTGTATCTTCCTTGGTTGG |  |  |
| E1C | F : ATTACCACAGAGGGGACAAGGG | 66 °C | 1426 bp |
|  | R : GCAAATCCAGTAGGACAGCAACA |  |  |
| E1D | F : TACAAGGGACTGTTGATGGGCTT | 63 °C | 1508 bp |
|  | R : TGAAAATAGAGTAGGAGGGAGGGG |  |  |
| E1E | F : CCCATCCCCATTCAACTTCC | 60 °C | 1355 bp |
|  | R : GCGACCTACACCACAGCCC |  |  |
| E1F | F : TTCCCACCTAACTCCCTTTTCCT | 66 °C | 1664 bp |
|  | R : ACAGTCCCAAATGCCCTCCC |  |  |
| E1G | F : GCCCTCTAACTTTTTACATTACCCC | 66 °C | 1706 bp |
|  | R : GCACAAGAACCCAGACAAATACATC |  |  |
| E1H | F : GCCTTGTCTTGGGACTGTTACTATG | 64 °C | 1455 bp |
|  | R : GTATGGGCTGCTGTTTGATGGA |  |  |
| E2 | F : CATCAAACAGCAGCCCATACTC | 63 °C | 1390 bp |
|  | R : ATCATTTCTAAGCCCTCACTTCAG |  |  |
| E2EL*^**^* | F : GGAGCATCAACCAGCCCC | 63 °C | 9538 bp (1868 bp) |
|  | R : GACACAGAAGCATACAAAGCACGA |  |  |
| ELB1 | F : TTTCGTGCTTTGTATGCTTCTGTG | 64 °C | 1014 bp |
|  | R : GAGTAAGGTGTTGCTGGCTGATG |  |  |
| ELS1 | F : GAAGCACCAAGACCAAGGGAA | 64 °C | 1535 bp |
|  | R : CCACAGGCACAACAACGAGG |  |  |
| ELB2 | F : TTCCTTCATTTCTTTCCTCTTACC | 60 °C | 1697 bp |
|  | R : AGTCTTTCATTCATCAGGCATTT |  |  |
| ELS2 | F : GTGAGGCAGGCATTATCTCTACAA | 60 °C | 980 bp |
|  | R : CCCATCTCGTCAATCAGGCA |  |  |
| ELB3 | F : CTTGCTCCTTTGCCTGATTG | 58 °C | 1071 bp |
|  | R : TTTCACTCCATTTCTTCTTACTGTTG |  |  |
| ELS3 | F : TGCCAGAGAGTCAGAAAGCCA | 58 °C | 1571 bp |
|  | R : GAAATGAGGGGGAACAAAGGAA |  |  |
| ELS4 | F : CAGAGAGGGTGGGATAGGAGGAAG | 58 °C | 860 bp |
|  | R : TGTTTCACATCAGTTCACAAGTCCA |  |  |
| β | F : CTCTTTCTTGAGGTGGGGGT | 64 °C | 860 bp |
|  | R : GCTCCTGCTTGGTAATGGG |  |  |
| *SRY*  (NM_214452.3) | F : CTGGGATGCAAGTGGAAAAT | 62 °C | 250 bp |
|  | R : GGCTTTCTGTTCCTGAGCAC |  |  |
| *GAPDH^**^* (NM001206359) | F : GGTCGGAGTGAACGGATTTG | 62 °C | 276 bp (174 bp) |
|  | R : GCCGTGGGTGGAATCATACT |  |  |
| *Primers were designed to result from BLAST analysis and the homologue region in sus scrofa 10.2 scafford sequence was used (GenBank acc. no. NW_003612825.1) except *SRY* and *GAPDH*.  *^**^*The primer sets were designed to span intron | | | |
|  |  |  |  |
| †The length in parenthesis represent the size of cDNA PCR amplicons | |  |  |
