# Supplementary material for: Identification of the Porcine XIST Gene and Its Differential CpG Methylation Status in Male and Female Pig Cells
Source: PLoS One. 2013 Sep 9;8(9):e73677. doi: 10.1371/journal.pone.0073677 (PMC3767593; doi:10.1371/journal.pone.0073677)
Supplement: Table S2 — List of restriction enzymes used for each amplicon and the predicted sizes of the digested fragments. (DOCX) [file pone.0073677.s007.docx]

| Table S2. List of restriction enzymes used for each amplicon and the predicted sizes of the digested fragments. | | | |
| --- | --- | --- | --- |
|  |  |  |  |
| Amplicon | Restriction enzyme | Predicted amplicon size (bp) | Digested fragment size (bp)^*^ |
| E1S3 | SacII | 799 | 144 + 655 |
| E1S1 | BstUI | 953 | 686 + 267 |
| E1B1 | EcoRI | 899 | 599 + 300 |
| E1S2 | EcoRI | 840 | 199 + 641 |
| E1S4 | DraI | 1429 | 208 + 849 + 372 |
| E1N1 | EcoRV | 1512 | 418 + (1094 + ≈500) |
| E1N2 | DraI | 1084 | 159 + (≈400) + (925+≈200) |
| E1A | BamHI | 1649 | 989 + 660 |
| E1B | ScaI | 1676 | 1189 + 487 |
| E1C | ScaI | 1426 | 296 + 675 + 455 |
| E1D | SpeI | 1508 | 715 + 793 |
| E1E | EcoRV | 1355 | 783 + 572 |
| E1F | ScaI | 1664 | 1313 + 351 |
| E1G | DraI | 1706 | 848 + 250 + 608 |
| E1H | ScaI | 1455 | 1112 + 343 |
| E2 | ScaI | 1390 | 401 + 989 |
| ELB1 | EcoRV | 1014 | 471 + 543 |
| ELS1 | SpeI | 1535 | 524 + 1011 |
| ELB2 | ScaI | 1697 | 157 + 1540 |
| ELS2 | SpeI | 980 | 286 + 694 |
| ELB3 | ScaI | 1071 | 728 + 343 |
| ELS3 | HindⅢ | 1571 | 638 + 933 |
| ELS4 | EcoRV | 860 | 535 + 345 |
| ^*^ ‘≈’ indicates the approximate size suspected by comparison with DNA marker. Because the E1N1 and E1N2 contain gap-sequence, the digested DNA fragments were different to expected fragments lengths. | | | |
