# Supplementary material for: Identification of the Porcine XIST Gene and Its Differential CpG Methylation Status in Male and Female Pig Cells
Source: PLoS One. 2013 Sep 9;8(9):e73677. doi: 10.1371/journal.pone.0073677 (PMC3767593; doi:10.1371/journal.pone.0073677)
Supplement: Table S3 — List of primer pair sequences for 5`- and 3`- RACE-PCR. (DOCX) [file pone.0073677.s008.docx]

| Table S3. List of primer pair sequences for 5`- and 3`- RACE-PCR | | | |
| --- | --- | --- | --- |
| Primer | | Sequence (5` → 3`) | Tm (°C) |
| 5`-Race | RT primer | ^*^p-ACAAGTAGCCCTCAG | 50°C |
|  | 1st round | F: GTTGGGTTTTGTGGTTCGTT | 54°C |
|  |  | R: GTTGGAGAAAGAGGGGGACA |  |
|  | 2nd round | F: TGAGTGGACCTACGGCTT | 56°C |
|  |  | R: CCGATGGGCATAATACACA |  |
| 3`-Race | 1st round | F: GATATCAGCTGGATGCAGTTATTC | 55°C |
|  |  | R: CTGATCTAGAGGTACCGGATCC^†^ |  |
|  | 2nd round | F: TCTTTCTTGAGGTGGGGGTG | 60°C |
|  |  | R: CTGATCTAGAGGTACCGGATCC^†^ |  |
| ^*^RT-primer for 5`-RACE was phosphorylated at the 5`-end | | | |
| ^†^The reverse primer for 3`-RACE-PCR was kit supplied; both the 1st and 2nd round PCR was performed with the same reverse primer. | | | |
