# Supplementary material for: Identification of the Porcine XIST Gene and Its Differential CpG Methylation Status in Male and Female Pig Cells
Source: PLoS One. 2013 Sep 9;8(9):e73677. doi: 10.1371/journal.pone.0073677 (PMC3767593; doi:10.1371/journal.pone.0073677)
Supplement: Table S4 — List of primer pairs for bisulfite sequencing. (DOCX) [file pone.0073677.s009.docx]

| Table S4. List of primer pairs for bisulfite sequencing | | | |  |  |
| --- | --- | --- | --- | --- | --- |
| Primer | | Sequence (5` → 3`) | Tm (°C) | Region^†^ | Size (bp) |
| BS1 | Outer | F: TGGTTAAATGAGGTATTTGGA | 54°C | -425 ~ +100 | 525 |
|  |  | R: CCATAAAACATAACTAAAAACTAAA |  |  |  |
|  | Inner (BS1-1) | F: TTTGTTATATTGTTTGTGGAAAA | 50°C | -329 ~ +100 | 429 |
|  |  | R: CCATAAAACATAACTAAAAACTAAA |  |  |  |
| BS2 | Outer | F: TTGGGATATTTTAAGGTAATTTT | 56°C | +206 ~ +792 | 587 |
|  |  | R: ATTTTATAAACATTCCAAACAATACA |  |  |  |
|  | Inner 1 (BS2-1) | F: GGGATATTTTAAGGTAATTTTT | 56°C | +208 ~ +557 | 350 |
|  |  | R: AAAAACAAATATCCATTACCCT |  |  |  |
|  | Inner 2 (BS2-2) | F: TTTATTAGGGTAATGGATATTTG | 56°C | +530 ~ +792 | 263 |
|  |  | R: ATTTTATAAACATTCCAAACAATACA |  |  |  |
| BS3^*^ | | F: TTGTATTGTTTGGAATGTTTATA | 54°C | +766 ~ +1146 | 381 |
|  |  | R: CCTTCTCCCTCTCATTTTCT |  |  |  |
| BS4 | Outer | F: AGAAAATGAGAGGGAGAAGGTT | 58°C | +1127 ~ 1795 | 634 |
|  |  | R: CCTTAAATACCACCCACTAAAAA |  |  |  |
|  | Inner 1 (BS4-1) | F: AGAAAATGAGAGGGAGAAGGTT | 60°C | +1127 ~ +1452 | 326 |
|  |  | R: TATCCACATAACAAAATCAACCA |  |  |  |
|  | Inner 2 (BS4-2) | F: AGGGATAATATGGTTGATTTTGTT | 52°C | +1420 ~ +1759 | 341 |
|  |  | R: CCTTAAATACCACCCACTAAAAA |  |  |  |
| ^*^BS3 was amplified without performing nested PCR. | | | | | |
| ^†^The region analyzed in this study spanned 2 kb upstream and downstream of one of the transcription start sites (289233^rd^ nucleotide of the pig X-chromosome scaffold sequence, NW_003612825.1) | | | | | |
