# Supplementary material for: Identification of the Porcine XIST Gene and Its Differential CpG Methylation Status in Male and Female Pig Cells
Source: PLoS One. 2013 Sep 9;8(9):e73677. doi: 10.1371/journal.pone.0073677 (PMC3767593; doi:10.1371/journal.pone.0073677)
Supplement: Material and method S1 — PCR amplification condition. (DOCX) [file pone.0073677.s010.docx]

**Supplementary Material and Method**

**Identification of the Porcine XIST gene and its differential CpG methylation status in male and female pig cells**

***Jae Yeon Hwang^1^, Eun Bae Kim^2, 3^, Hakhyun Ka^4^ and Chang-Kyu Lee^1^***

^1^*Department of Agricultural Biotechnology, Animal Biotechnology Major, and Research Institute for Agriculture and Life Sciences, Seoul National University, Seoul, Republic of Korea*

*^2^Department of Food Science and Technology, University of California, Davis, California, United States of America*

*^3^Department of Animal Products and Food Science, College of Animal Life Sciences, Kangwon National University, Chuncheon, Kangwon-do, Republic of Korea*

*^4^* *Division of Biological Science and Technology, Institute of Biomaterials and Institute for Poverty Alleviation and International Development, Yonsei University, Wonju, Kangwon-do, Republic of Korea*

* Correspondence and reprint requests:

Chang-Kyu Lee, PhD

Department of Food and Animal Biotechnology

College of Agricultural Life Science, Seoul National University

Seoul, 151-921 Korea

Tel: +82-2-880-4805

Fax: +82-2-873-4805

E-mail: leeck@snu.ac.kr

**PCR amplification condition**

**RT-PCR and genomic DNA PCR condition**

PCR was performed according to the following conditions: 1 cycle of 95°C for 7 min; 35 cycles of 95°C for 45 sec, annealing at the temperature listed in Table S1 for 30 sec, and 72°C for 1 min 30 sec; and 1 cycle of 72°C for 10 min. Amplification of *SRY* and *GAPDH* primer pairs (Table S1) was performed according to the following conditions: 1 cycle of 95°C for 5 min; 30 cycles of 95°C for 30 sec, 60°C for 30 sec, and 72°C for 45 sec; and 1 cycle of 72°C for 7 min.

**RACE-PCR of 5’- and 3’- region**

For the 5’ RACE PCR, the first round of PCR was performed according to the following conditions: 1 cycle of 95°C for 7 min; 54°C for 5 min; 72°C for 40 min; 30 cycles of 95°C for 40 sec, 54°C for 1 min 30 sec, and 72°C for 2 min; and 1 cycle of 95°C for 40 sec, 54°C for 1 min 30sec, and 72°C for 15 min. The second round of PCR was carried out as follows: 1 cycle of 95°C for 7 min; 30 cycles of 95°C for 35 sec, 56°C for 30 sec, and 72°C for 1 min; and 1 cycle of 72°C for 10 min.

In case of 3**’** RACE PCR, the first round of PCR was performed as follows: 1 cycle of 95°C for 5 min; 55°C for 5 min; 72°C for 40 min; and 20 cycles of 95°C for 40 sec, 55°C for 1 min, and 72°C for 2 min; and 1 cycle of 95°C for 40 sec, 55°C for 1 min, and 72°C for 15 min. Nested PCR was performed according to the following conditions: 1 cycle of 95°C for 5 min followed by 35 cycles of 95°C for 30 sec, 60°C for 30 sec, and 72°C for 1 min 30 sec.

**PCR for Bisulfite sequencing**

First-round PCR was performed using 1 cycle of 95°C for 5 min and 35 cycles of 95°C for 30 sec, annealing at the temperature listed in Table S4 for 30 sec, and 72°C for 2 min; and 1 cycle of 72°C for 10 min. Second-round PCR was performed as follows: 1 cycle of 95°C for 7 min; 40 cycles of 95°C for 35 sec, annealing temperature for 30 sec, and 72°C for 1 min: and 1 cycle of 72°C for 10 min. BS3 was amplified according to the following conditions: 1 cycle of 95°C for 5 min; 40 cycles of 95°C for 30 sec, 54°C for 30 sec, and 72°C for 2 min; and 1 cycle of 72°C for 10 min.
